# The complete mitochondrial genome of the hybrid grouper *Epinehelus_moara ♀* × *Epinephelus_lanceolatus ♂* with phylogenetic consideration

**DOI:** 10.1080/23802359.2016.1197071

**Published:** 2016-08-31

**Authors:** Lin Tang, Zhujing Tang, Xiao Chen, Jiaxing Chen, Shuisheng Li, Yun Liu, Yong Zhang, Haoran Lin

**Affiliations:** a State Key Laboratory of Biocontrol, Institute of Aquatic Economic Animals and Guangdong Provincial Key Laboratory for Aquatic Economic Animals, School of Life Sciences, Sun Yat-Sen University, Guangzhou, China;; b South China Sea Bio-Resource Exploitation and Utilization Collaborative Innovation Center, Guangzhou, China;; c Marine Fisheries Development Center of Guangdong Province, Huizhou, China

**Keywords:** *Epinehelus moara ♀* × *Epinephelus lanceolatus ♂*, mitochondrial genome, phylogenetic

## Abstract

The complete mitochondrial genome of the hybrid grouper *Epinehelus moara ♀* × *Epinephelus lanceolatus ♂* is presented in this study. The mitochondrial genome is 16,418 bp long and consists of 13 protein-coding genes, 2 rRNA genes, 22 tRNA genes and a control region. The gene order and composition of the hybrid grouper mitochondrial genome were similar to that of most other vertebrates. The nucleotide compositions of the light strand in descending order was 28.86% of G, 28.61% of T, 26.46% of A and 16.08% of C. With the exception of the NADH dehydrogenase subunit 6 (ND6) and eight tRNA genes, all other mitochondrial genes are encoded on the heavy strand. The phylogenetic analysis by maximum-likelihood (ML) method shows that the hybrid grouper has closer relationship to *Epinehelus moara* and *Epinehelus bruneus*.

*Epinehelus moara* and *Epinephelus lanceolatus* both belong to Serranidae in the order Perciformes. The hybrid grouper (*Epinehelus moara ♀* ×*Epinephelus lanceolatus ♂*) is inclined to take rock fish *E. moara* as female parent, with grouper *E. lanceolatus* as male parent of hybrid F1 generation. Both of them possess many characteristics, such as fast growth, enormous size (Heemstra & Randall 1993; Hseu et al. 2004). As there is little information about its genetic characteristics, the purpose of our study was to sequence the complete mitochondrial genome of the hybrid grouper (*Epinehelus moara ♀* × *Epinephelus lanceolatus ♂*) by using the next-generation sequencing (NGS) strategy (Xie et al. 2015), in order to develop new DNA markers for the studies on population genetics of the hybrid grouper. The specimen was obtained from Daya Bay Fishery Development Center, Guangdong, China. The total genomic DNA was extracted from the fin of the fresh fish using the salting-out procedure (Howe et al. 1997).

The complete mitochondrial genome of the hybrid grouper *Epinehelus_moara ♀* x_*Epinephelus_lanceolatus ♂* (GenBank accession number KX147236) is 16,418 bp in length, consisting of 13 protein-coding genes, 2 ribosomal RNA genes (12S rRNA and 16S rRNA), 22 transfer RNA genes (tRNA) and 1 control region (Figure 1), which is the same as the typical vertebrates (Wang et al. 2008). Most of the genes are encoded on the heavy strand, with only the NADH dehydrogenase subunit 6 (ND6) and eight tRNA genes [Gln, Ala, Asn, Cys, Try, Glu, Pro, Ser (TGA)] encoded on the light strand. Overall nucleotide compositions of the light strand are 26.46% of A, 16.08% of C, 28.61% of T and 28.86% of G. However, the most representative base is G and the bias against A was observed, which is different from the base compositions of mitochondrial genome of other teleosts.

**Figure 1.**
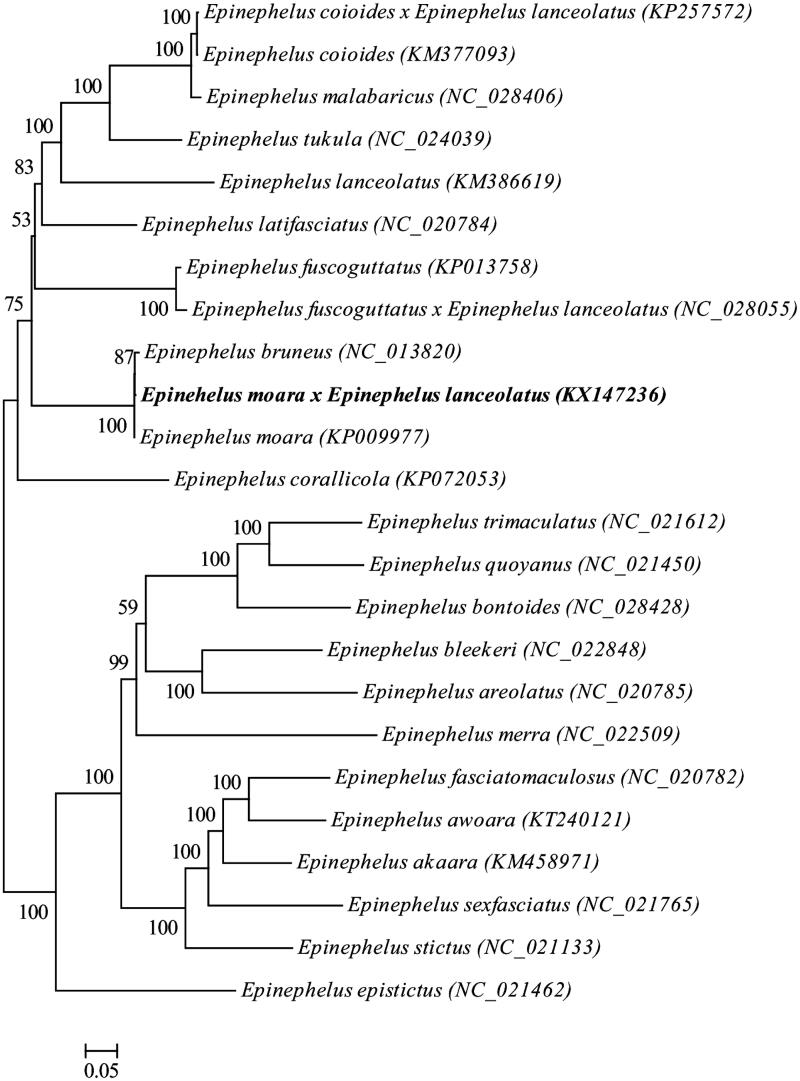
The ML phylogenetic tree of *Perciformes species*. Numbers on each node are bootstrap values of 100 replicates.

All the protein-coding genes begin with an ATG start codon except for COX1, ND4 started with GTG and ATP6 started with CTG. Four types of top codons revealed are TAA (COX1, ATP8, ATP6, ND4L, ND6, ND1), T (ND2, COX2, ND3, ND4, CYTB) and TAG (ND5). The 12S and 16S rRNA genes are located between the tRNA-Phe (GAA) and tRNA-Leu (TAA) genes, and are separated by the tRNA-Val gene with the same situation found in other vertebrates. Most genes are either abutted or overlapped. The 22 tRNA genes vary from 69 to 76 bp in length. All these could be folded into a typical cloverleaf secondary structure although numerous non-complementary and T–G base pairs exist in the stem regions. The control region was 998 bp in length, located between tRNA-Pro (TGG) and tRNA-Phe (GAA) gene.

The phylogenetic position of the hybrid grouper *Epinehelus_moara ♀ × Epinephelus_lanceolatus ♂* was reconstructed with the complete mtDNA sequences from 24 species of Perciformes by using the maximum-likelihood (ML) methods (Kumar et al. 2004). As shown in Figure 1, the hybrid grouper *Epinehelus_moara ♀* x*_Epinephelus_lanceolatus ♂* has closer relationship to *Epinehelus_moara* and *Epinehelus bruneus*.
